# Gene therapy as a potential therapeutic option for Duchenne muscular dystrophy: A qualitative preference study of patients and parents

**DOI:** 10.1371/journal.pone.0213649

**Published:** 2019-05-01

**Authors:** Holly Landrum Peay, Ryan Fischer, Janice P. Tzeng, Sharon E. Hesterlee, Carl Morris, Amy Strong Martin, Colin Rensch, Edward Smith, Valeria Ricotti, Katherine Beaverson, Hannah Wand, Carol Mansfield

**Affiliations:** 1 Center for Newborn Screening, Ethics, and Disability Studies, RTI International, Research Triangle Park, North Caroilina, United States of America; 2 Parent Project Muscular Dystrophy, Hackensack, New Jersey, United States of America; 3 Lion Therapeutics, Asklepios BioPharmaceutical, Inc., Research Triangle Park, North Carolina, United States of America; 4 Solid Biosciences, Cambridge, Massachusetts, United States of America; 5 Center for Duchenne Muscular Dystrophy at UCLA, University of California Los Angeles, Los Angeles, California, United States of America; 6 Duke University School of Medicine, Durham, North Carolina, United States of America; 7 Rare Disease Research Unit, Pfizer, Inc, Cambridge, Massachusetts, United States of America; 8 Stanford Healthcare and ClinGen, Sanford, California, United States of America; 9 RTI Health Solutions, RTI International, Research Triangle Park, North Carolina, United States of America; University of Tennessee Health Science Center, UNITED STATES

## Abstract

**Objectives:**

Duchenne muscular dystrophy (DMD) is a rare neuromuscular disorder that causes progressive weakness and early death. Gene therapy is an area of new therapeutic development. This qualitative study explored factors influencing parents’ and adult patients’ preferences about gene therapy.

**Methods:**

We report qualitative data from 17 parents of children with DMD and 6 adult patients. Participants responded to a hypothetical gene therapy vignette with features including non-curative stabilizing benefits to muscle, cardiac and pulmonary function; a treatment-related risk of death; and one-time dosing with time-limited benefit of 8–10 years. We used NVivo 11 to code responses and conduct thematic analyses.

**Results:**

All participants placed high value on benefits to skeletal muscle, cardiac, and pulmonary functioning, with the relative importance of cardiac and pulmonary function increasing with disease progression. More than half tolerated a hypothetical 1% risk of death when balanced against Duchenne progression and limited treatment options. Risk tolerance increased at later stages. Participants perceived a ‘right time’ to initiate gene therapy. Most preferred to wait until a highly-valued function was about to be lost.

**Conclusion:**

Participants demonstrated a complex weighing of potential benefits against harms and the inevitable decline of untreated Duchenne. Disease progression increased risk tolerance as participants perceived fewer treatment options and placed greater value on maintaining remaining function. In the context of a one-time treatment like gene therapy, our finding that preferences about timing of initiation are influenced by disease state suggest the importance of assessing ‘lifetime’ preferences across the full spectrum of disease progression.

## Introduction

Duchenne muscular dystrophy (Duchenne) is a rare neuromuscular disorder with onset in early childhood. The progressive muscle weakness leads to loss of motor function that is further complicated by pulmonary and cardiac complications, leading to death typically in the third decade [1–3]. The distress associated with the Duchenne prognosis, progressive loss of function, and limitations in activities of daily living impact the quality of life of patients [3–5] and their caregivers [6–13].

Most patients with Duchenne face high unmet medical need. There are two approved therapies in the United States: a corticosteroid therapy appropriate for all patients that slows progression of muscle degeneration [14–15] and a mutation-specific therapy indicated for less than 15% of patients [16–18]. Active drug development of multiple investigational therapeutic modalities continues in Duchenne [19], including in gene therapy (also termed gene transfer) [20,21].

The first phase I gene therapy trial in Duchenne took place in 2002, when nine patients received intramuscular injections of a plasmid containing full-length dystrophin c-DNA [22]. Subsequently, in 2004 a hybrid adeno-associated viral vector containing a miniaturized version of the dystrophin gene was injected into six volunteers with Duchenne. The results demonstrated modest evidence of gene transfer, but also pre-existing immune response to the dystrophin protein [23]. Trials for limb-girdle and Becker muscular dystrophies have progressed with, respectively, regional delivery of viral vectors to a single limb [24] and multiple injections within a single muscle [25]. Evidence from animal models suggest that the introduced dystrophy gene can last at least eight years after gene transfer and may lead to long-term stabilization in muscle function [20, 26, 27]. In this promising context, several companies anticipating and initiating clinical trials partnered with Parent Project Muscular Dystrophy (PPMD) to assess patient and caregiver preferences about the therapeutic application of gene therapy technologies.

PPMD and the Duchenne community have been on the cutting edge of rare-disease patient-focused drug development (PFDD) in Duchenne [28–30]. PPMD, RTI International and an advisory committee of stakeholders developed the current study to explore knowledge, preferences, and intentions regarding gene therapy as a therapeutic option for treating Duchenne muscular dystrophy. There are no existing data on patient or parent attitudes or preferences for gene therapy. This exploratory qualitative interview study is part of a larger gene therapy preference initiative. In addition to supporting quantitative instrument development in the next phase of the initiative, this interview study was developed to provide a rich account of the factors that underlie our participants’ perceptions and preferences regarding gene therapy, support hypothesis generation for future studies, and inform educational efforts.

## Materials and methods

Consistent with our previous PFDD work [30, 31], study decision making occurs through a consensus-driven process facilitated through an advisory committee comprising a patient representative, a caregiver representative, an expert clinician, and representatives from industry. The committee advised on the development of the instrument, discussed results interpretation and reporting, and participate as authors. Study data is owned by PPMD.

Eligible participants were parents of children (of any age) with Duchenne and adults with Duchenne. Participants (or participants’ children with Duchenne) had not participated in gene therapy trials. PPMD staff conducted study recruitment using their existing databases to directly contact potential participants via email and newsletter recruitment sent to eligible members of the Duchenne Registry (www.DuchenneRegistry.org) and via PPMD’s website and advocacy email contact lists. Those interested gave permission to be contacted by RTI staff to schedule interviews. Interviews were conducted by telephone by an RTI researcher experienced in qualitative research. Each interview lasted approximately 30–45 minutes. We continued recruitment of parent participants until our continual review of interviewer notes and transcripts identified saturation [32], e.g., when no new themes were identified; this occurred after the fifteenth interview. We interviewed and coded two final interviews as a quality check. We recruited patient participants until the pre-determined close of the recruitment period. We achieved saturation in the adult cohort on primary themes but may have attained additional nuance in our thematic interpretation with more participants; this is later referenced as a limitation.

Participants received a consent statement prior to the interview, and the interviewer reviewed the consent at interview initiation. The interview began with a minimally-prompted assessment of participant’s baseline knowledge and attitudes. During this phase of the interview, the interviewer did not provide any information about gene therapy or correct any misconceptions; corrections were done at the end of the interview, as needed. The interviewer then employed a vignette (Table 1) developed with expert advisory input as a conservative representation of gene therapy in Duchenne. The final portion of the interview utilized a vignette of being invited to join a gene therapy trial. We closed with a debrief about the hypothetical components and participants were offered educational materials. These efforts were intended to reduce the risk of the interview acting as an unintended intervention. All interviews were recorded and transcribed to ensure accuracy in documenting participants’ responses. During and immediately after each interview, the interviewers completed a descriptive interview note for each participant.

**Table 1:** Gene therapy vignette.

Anticipated benefits	Variable benefit based on time of initiation, with greater benefit at earlier stages of progression
	Potential for stopping progression and some gain of function
	Impact on skeletal muscle, breathing, and heart function
Mode of administration	One-time IV administration
“Caveats” of gene therapy	Duration of expected benefit: 8–10 years; insufficient evidence regarding longer-term benefit
	Single administration due to risk of immune response
	Ineligibility for most future clinical trials
Risks	Described as serious immune response soon after infusion
	Risk of death initially described as 1 in 100; subsequently described as 1 in 100,000

### Analysis

We conducted qualitative analysis using NVivo version 11 to query and code each of the transcripts for descriptive and interpretive components. A subset of interviews and associated interview notes were first reviewed by two experienced investigators (HLP and JPT) to identify and organize the coding domains, which informed the development of an analysis codebook comprising a set of unique and well-defined codes. After refining the coding domains, one investigator (JPT) applied the codes to each of the interview transcripts in NVivo. Two investigators (HLP and JPT) examined the coding reports and discussed and reconciled discrepancies in the coding, and then began thematic analysis through the identification of coding categories. Those categories were discussed by the authoring group to identify patterns, unexpected and divergent findings, and the emergence of additional interpretive themes. We assessed concordance of these themes with interviewer notes as a final quality check. The authoring group discussed the interpretations and implications of the data.

### Human subjects determination

The study was determined to be exempt from IRB review by the RTI Institutional Review Board.

## Results

### Participants

Interviews were conducted between March and May 2017. The sample consisted of six adults with Duchenne ages 21–26, and 17 parents of children with Duchenne ranging from ages 5–32. All adults with Duchenne were male, and all used wheelchairs fulltime. Four reported needing some help with self-care and two reported needing a lot of help.

Of the parent participants, three were fathers and 14 were mothers. Three participants had two children with Duchenne (20 total affected children). Eleven children walked independently, eight used wheelchairs full time, and one was in bed full time. Parents reported that nine children do most or all self-care independently, eight need some help, and three need a lot of help.

### Qualitative results

We present qualitative results in the order in which the questions were posed and answered, beginning with baseline knowledge and attitudes and then progressing through the vignette components and ending with participants’ own interest in gene therapy trials. Participants typically used “younger” to describe people with less severely progressed Duchenne, and as age is highly correlated with ambulation and function, we employ the same convention for simplicity.

#### Baseline knowledge and attitudes about gene therapy

Although participants expressed optimism, to varying degrees, about gene therapy as a treatment option for Duchenne, both parent and adult respondents reported mixed attitudes about how realistic gene therapy seems to them. Most participants expressed guarded enthusiasm due to concerns about the long research timeline in relation to the progressive nature of Duchenne. Parents and adults were concerned about the considerable uncertainty related to the amount of benefit to be expected, and about the types of and probability of risks. Several participants expressed confusion about eligibility for gene therapy and concern about whether gene therapy could work for those with a range of different genetic mutations. A few were uncertain or skeptical about the ability to deliver the gene therapy product into muscle cells all over the body.

#### Response to vignette: Meaningful gene therapy-associated benefits

In response to the description of benefit provided in the vignette, all respondents placed high value on the benefit of stabilization in skeletal muscle functioning. The possibility of a gain of additional muscle function was perceived as an even stronger benefit. Maintaining heart and lung function were high priorities given their role in mortality.

***But the heart and lungs are the weak spot*, *and it’s not the disease that kills these boys*. *It’s the risk of pneumonia and the common cold and the simple things that take them from us so early*. *So yes*. *Heart and lung function is A one*, *number one priority***.

***[Parent 9]***

***I think the heart and lungs*. *That’s most important for me right now because it’s about the vital organs***.

***[Adult 2]***

The potential benefits were valued based on association with improved quality of life, maintaining independence in activities of daily living, and extending lifespan. Most participants described weighing the potential benefits of gene therapy against the limited therapeutic options currently available for most patients living with Duchenne.

There were some differences in responses based on Duchenne progression. Parents of younger children placed a higher value on skeletal muscle functioning to preserve mobility, encourage independence, and improved peer interactions. Parents of younger children acknowledged that heart and lung functioning would eventually become the greater priority.

Parents of older children described the muscle benefits as important in preserving self-care and improving quality of life, and most reported that pulmonary and cardiac benefits were even more important. Parents with older children reported that as their child’s function declines their therapeutic options also diminish, resulting in willingness to accept a smaller but still meaningful benefit.

Adult participants described the value of stopping their decline and preserving their ability to conduct self-care activities and maintain independence. The two oldest respondents focused on stabilization while the younger adults focused on adding function, maintaining strength, or maintaining the ability to take care of themselves. Participants describe valuing these benefits in relation to having fewer treatment options as they get older.

***And I’m right on the verge of possibly losing ability to use the bathroom myself or eat myself or drink myself*, *and maintaining ability to do that would be really*, *really huge for me***.

***[Adult T51]***

#### Response to vignette: Limitations and caveats of gene therapy

**Mode of Administration**. Most participants were not concerned about IV administration, though it was perceived negatively by a few parents and adults based on fear or dislike of needles. There were no apparent differences in preferences based on Duchenne progression.

#### Time-limited benefit

Parents reported a range of attitudes about the potential for time-limited benefit. A few parents found the limited treatment duration difficult to understand and accept. Respondents who were more accepting tended to focus on benefits that could accrue over that time period or were optimistic about novel treatment options or a different vector becoming available before the benefit associated with gene therapy ended.

***I think we would still be willing to try it*, *and hope and pray that in the next eight to ten years something else comes up***.

***[Parent 16]***

Some parents viewed the time-limited benefit in a positive frame when juxtaposed against the limited lifespan of patients with Duchenne and the limited treatment options available (particularly parents of further progressed children).

***Eight to ten years for anybody is more than what they have right now***.

***[Parent 9]***

Given the limited treatment duration, other parents preferred to wait to use gene therapy until they perceived that their child absolutely needed the treatment. These respondents were typically parents with younger children, or parents of older children reflecting on what they might choose if they had younger affected children.

***To me it would make more sense if you were gonna get more bang for your eight to ten year buck*, *it’d be better to wait a little bit longer*. *So based on where my kids were*, *I would say age seven to eight would be more appropriate to wait***.

***[Parent 5]***

***I guess my immediate thought is then we would not administer that as a toddler*. *It’d be administrated maybe when they’re starting to lose the ability to walk***.

***[Parent A14]***

Adult participants also reported mixed attitudes regarding the time-limited benefit. Respondents who were more accepting brought up the potential treatment benefits and lack of other options. Four anticipated more treatment options or the availability of a ‘booster’ within the next 8–10 years. Others opted to wait until they absolutely needed the treatment.

***Eight to ten years is a lot because it’s different when they’re younger*, *it hasn’t progressed as much*. *If you get eight to ten years to stop the progression when you’re young*, *that’s enormous because a lot of the progression [occurs] when the skeletal muscle turns to scar tissue*, *like it can’t come back***.

***[Adult C53]***

***I guess you could use it if there’s anything like major going on*. *You know*, *use it like maybe not a last ditch effort*, *but maybe to add to your life a little bit* …*eight years is a ton*. *There could be more stuff available by that time then***.

***[Adult 5]***

#### Limit to one-time administration

Several parent participants had a negative reaction to and/or disbelief about the one-time administration. Further, several worried about the implications for a patient for whom the therapy had no benefit upon first administration. These parents valued maintaining treatment options to the full extent possible.

***If the treatment is given to him one time*, *I would do it obviously if it showed a benefit*, *but I guess the question would be what if his body rejects or doesn’t accept the treatment*? *Is there a backup plan*? *Is there another option*? *You know*, *do all the cells in the body accept it*? *Are we just kinda shooting in the dark*, *and giving him an IV*, *and seeing which cells in his body will accept***?

***[Parent 9]***

Other parents and adult participants described being neutral or tolerant of the limitation, particularly when weighed against the potential of improved quality of life or preserved functioning. Others perceived the one-time administration to decrease the treatment burden, especially in relation to other approved or potential Duchenne therapies that require more frequent administration.

#### Ineligibility for future clinical trials

Most parent participants expressed that ineligibility for future trials was a considerable concern that was compounded by the uncertain benefits of gene therapy. Parents with older children, however, were less concerned because most current experimental therapies target a younger population.

***It’s scary for a parent because what if the body did not accept that particular drug*? *You didn’t even go through the whole trial*. *And if you were to say that*** “***Okay*. *Because you did that*, *you cannot come to this trial***.” ***That is not acceptable in my mind***.

***[Parent 12]***

Two adult respondents had a negative reaction to loss of eligibility due to their optimism about future therapeutic options. The other respondents were not particularly concerned due to their perception that there were few or no trial options available to them.

#### Response to vignette: Risk perception and risk tolerance

Participants were asked to respond to a risk scenario where 1 in 100 people who use gene therapy die soon after infusion due to massive immune response. **Slightly more than half of the parents (n = 9 of 17) reported being tolerant of the 1% risk of death**. They described this as “pretty good odds” when balanced with the benefits and the inevitable Duchenne progression without new treatments. Many were parents of older children. When asked to describe an unacceptable risk of death, parent participants reported risks that ranged from 10% to 50%.

***I guess that’s sad*, *but it’s a risk in life unfortunately*. *And there’s so much that can be gained*, *but it sounds like***…***you have better odds with that than you do with not doing anything***.

***[Parent 10]***

#### Slightly less than half of parents (n = 7 of 17) perceived the 1% risk as highly worrisome or unacceptable

These parents tended to have younger children. Some perceived being at greater risk of falling into the 1% with the adverse outcome due to already having experienced a rare event in having a child with Duchenne. Several questioned whether risk mitigation could reduce the chance of death.

***I feel like we’re always that one percent chance one***… ***my point it’s like one percent*, *yes*, *but when you’re the one percent*, *it’s not a good feeling*. *I think we would still take the risk just because of how brutal this disease is*, *you know***.

***[Parent S15]***

**One parent (n = 1 of 17) could not determine his/her tolerance** for the 1% risk throughout the interview. The participant preferred to defer the choice to what his/her child would choose.

***You know*, *I think life’s a risk*. *I don’t know what I would do*. *I don’t know what my son would choose to do***….

***[Parent B18]***

Respondents concerned about the 1% risk described a 1 in 100,000 risk as more acceptable. Several reported that their risk perception was influenced by the progressive and fatal nature of Duchenne. This context caused most parents to be more risk tolerant, but several others to be more risk adverse.

Several parents described a desire to wait until their child was mature enough to participate in the decision about how much risk to accept. Many parents described how their tolerance for risk would increase as their children age, the child’s health status declines, and treatment options become more limited.

***I mean*, *that’s of course less riskier*, *but anytime you mention death* …*I mean*, *our boys are already at risk of that*. *So we don’t want to increase those chances I guess you could say***.

***[Parent A14]***

***In no way I’m suggesting that gene therapy should be tried first on the older Duchenne population*, *but at the same time the appetite for risk is definitely higher in that population than it would be in a younger population***.

***[Parent K11]***

***If my son is where he is right now*, *and someone told me twenty percent*, *I wouldn’t do it*. *If my son was in his late teens*, *and the progression is what natural history suggests*, *I would do it***.

***[Parent 12]***

**Most adult respondents (n = 5 of 7) described a 1% risk of death as “a reasonable risk”** when weighed against potential treatment benefits, though most demonstrated some risk-associated worry. Unacceptable levels of risk for adult respondents ranged from 15% to 50%. **One adult with Duchenne reported that he would not accept the 1% risk of death (n = 1 of 7) and one was unsure (n = 1 of 7)**. All adult participants (n = 7) were willing to accept the 1 in 100,000 risk of death.

***If it’s one percent*, *yeah*. *I think that would definitely cause me to like think twice*, *especially think very hard about whether it’s worth it***.

***[Adult 2]***

***I guess a reasonable risk*, *or something that’s not extreme*, *but does give a little tiny pause*, *but not a huge pause***.

***[Adult T51]***

Like parent participants, most adult respondents anticipated that their tolerance for risk may increase as their health status declines and their perceived near-term risk of death increases.

***If you’re older with DMD*, *then you still face those risks even if you don’t take [gene therapy]***…. ***If you’re younger*, *it’s an even bigger risk because you can survive eight to ten years without taking [gene therapy]*** …***As you get older*, *then you can tolerate higher risk***.

***[Adult 2]***

#### Interest in clinical trials for gene therapy

Sixteen of our parent participants were queried about their interest in having their child(ren) participate in a clinical trial using gene therapy; one participant was not asked this question during the interview. Regarding trial interest:

**Eight parents (8:16) expressed interest** in participating in a trial. They appreciated possible benefits, especially given the dearth of other options. But to make an informed benefit/risk determination, these parents also expressed a desire for information about anticipated benefits, risks, risk migration efforts, and logistical requirements of participating in the trial. A few questioned their child’s eligibility due to participation in other trials or advanced Duchenne progression. Although several indicated willingness to accept more risk as their child’s illness progressed, they noted that it may not be worth considering if their child is not eligible or healthy enough to participate.

***If there was a possibility of him extending life eight to ten years*, *plus the drug that he’s on right now*, *and this would give us more time for something*. *I would definitely consider it I believe*. *We would seriously consider doing it***.

***[Parent 13]***

**Six parents (6:16) indicated considerable hesitancy** about gene therapy trial participation. They expressed concerns about insufficient benefits given the potential risks and uncertainty, ineligibility for future clinical trials, and worries about the current health of their child. Parents of younger children and parents of adults with poor health status seemed to have more concerns about the benefit/risk balance.

***There’s so many question marks with the risks*. *I know there’s people that will try anything*, *but some of us are a little more cautious*. *So I think at this point we’re more guarded*. *We’re a little more cautious*. *You know*, *like I said*, *because he is so young***.

***[Parent 3]***

**One parent (1:16) had no interest in the trial** and expressed a preference for holding out until a cure became available. **One parent (1:16) reported not having an answer** regarding his/her trial interest.

**Among adult participants**, all 7 adults expressed interest in learning more about gene therapy trials. **Most adults (n = 5 of 7) reported favorable attitudes toward participation** if trials were available to them. Like parent participants, they were influenced by the lack of available treatment options but also stressed the need to learn more about benefits and harms. Several were concerned about the limitation of one-time dosing, and thus preferred to participate in a later-phase clinical trial. **Two adult participants (n = 2 of 7) shared serious trial-related concerns** that protocol demands and side effects may be too much for them, given their current health status.

***I think if I was eligible for it*, *then I think it would depend whether it’s stage one or stage two or stage three trial*, *and yeah*. *I think it would depend on if I could use it again*. *Like if it’s a phase one trial just to see if my body can tolerate it*, *and I can’t do it again*, *then yeah*. *That would change my thinking***.

***[Adult 2]***

***I’m really wary of ever doing a trial because I’m always worried about the complications and the side effects* … *for me I’m not really into clinical trials*. *I think they’re a great thing for the right people*, *but I’m kinda trying to avoid all those complications* …*I would want only to use it once it’s been proven to work***.

***[Adult D54]***

## Discussion

Our findings indicate cautious optimism about gene therapy as a therapeutic option for Duchenne. Our parent and adult participants placed high value on what they perceived as meaningful benefits to function and quality of life. The potential benefits were valued somewhat differently based on disease progression. Benefits to muscle function were important to maintaining or improving mobility in younger children, versus maintaining or improving independence and activities of daily living in older children and adults with Duchenne. Similarly, both groups valued improved cardiac and pulmonary function due to associated lifespan and quality of life benefits. Those in older age groups describe higher relative importance of cardiac and pulmonary benefits compared to muscle benefits.

When presented with a conservative, hypothetical gene therapy vignette, our participants demonstrated a complex weighing of the potential benefits against the potential harms, the lack of other treatment options, and the inevitable decline in untreated people with Duchenne. Many, but not all, participants reported a willingness to trade a 1% risk of treatment-related death for the anticipated benefits, even given one-time administration with time-limited benefits. For some participants, this likely reflects their optimism in the research enterprise, which reduced their concerns about the limitations presented in the vignette. A similar therapeutic optimism is reflected in the negative perceptions of many participants about loss of eligibility for future trials, especially among parents of younger children.

These findings, which are the first focused on gene therapy for Duchenne, expand upon prior studies of clinical trial preferences and attitudes. Prior studies found similar high interest and intentions to participate in non-gene-therapy trials, general optimism regarding scientific advancement juxtaposed with worry about disease progression, and concerns about access to new therapeutics under trial [33–36]. While two prior studies assessed tolerance for serious but non-fatal adverse trial events [37, 38], this is the first study to explore tolerance for risk of therapy-related death. In addition, it is the first to assess attitudes about a thearapy with limited benefit durability.

A compelling finding in this study that has not been previously reported was that participants perceived a ‘right time’ for use of gene therapy. This optimum time varied among participants. As this theme emerged, we obtained additional nuance from participants about their preferences for timing. Parents with younger children were more interested in waiting until their child’s functioning begins to decline. Similarly, when parents of older children imagined using gene therapy for a toddler, most indicated preferring to wait until the child was older. In contrast, two parents would not delay the use of gene therapy if available to a toddler, so the young child could keep up with peers and defer the need to handle motor decline until they are older and more mature. Our adult participants also described a ‘right time’ for gene therapy, often indicated at a point at which a valued function is about to be lost. Two respondents added that they might have preferred to use gene therapy when they were younger and had better functioning.

These data support a dynamic, multi-dimensional deliberation that is responsive to nuance in how benefits, risks, and limitations are described to participants. Our findings support the importance of measuring preferences and risk tolerance at different stages of disease. Given the current science of gene therapy, uncertainty is inherent in any descriptions of benefits and harms, and our participants referenced the impact of uncertainty on their attitudes. Future research should further address the role of uncertainty in the appraisals and preferences of patients and parents, and continue to assess preferences as emerging clinical trial data cause uncertainties to diminish.

### Limitations

Our sample has limitations in recruitment through an advocacy organization, which likely resulted in some bias and reduction in heterogeneity. In this exploratory study, however, our advocacy-engaged participants may represent those most likely to be recruited into gene therapy clinical trials. Our adult cohort was small; while we achieved saturation on primary themes, we may have obtained additional nuance with more participants. Participants responded to a hypothetical vignette with pre-defined attributes that did not address many important aspects of treatment decisions, such as access and cost; nor did the study address all preference-sensitive components to trial decisions, such as the use of a placebo-controlled design or the requirement for unpleasant procedures such as muscle biopsy. We will explore those attributes in a future quantitative preference survey as the next step in this project.

### Study implications

Our findings suggest specific educational needs related to gene therapy, including sources and types of uncertainty in benefits and risks. Our participants expressed a desire to better understand anticipated limitations such as one-time dosing and time-limited benefit, and the likelihood these limitations will be overcome over time. PPMD has begun creation of an educational initiative to meet the needs anticipated by and identified in this study.

Our data may be relevant to trial design, the informed consent process, and regulatory benefit/risk determinations. Our participants demonstrated a thoughtful weighing of benefits and harms through the lens of their current functioning, as well as their anticipation of future loss of function. Further, our participants demonstrated variation in risk tolerance. Many, but not all, participants indicated increased risk tolerance with illness progression as the value placed on maintaining function increased. The strong value placed on cardiac and respiratory benefits have implications for how the potential benefits of gene therapy are described to those recruited to clinical trials, and for outcomes measured in clinical trials. In the context of a one-time treatment like gene therapy, our finding that preferences about timing of initiation are influenced by disease state suggest the importance of measuring ‘lifetime’ preferences across the full spectrum of disease progression. Our group’s next step is to use the data collected in this study to inform the development of a quantitative patient preference survey that allows a nuanced assessment of maximum acceptable risk across a range of important functional time points in the lives of patients and parents living with Duchenne.
